# Nutritional and phytogenic modulation of caprine physiology: a systematic review of *Moringa oleifera* effects on metabolism, reproductive function, and lactation performance

**DOI:** 10.3389/fnut.2026.1820358

**Published:** 2026-04-15

**Authors:** Raza Mohai Ud Din, Nasir A. Ibrahim, Salwa Eman, Ahmed A. Saleh, Rahmat Ali, Mukesh Kumar, Mudathir Y. Abdulrahman, Nosiba S. Basher, Walid Elfalleh, Mohamed Osman Abdalrahem Essa, Hosameldeen Mohamed Husien, Mengzhi Wang

**Affiliations:** 1 Laboratory of Metabolic Manipulation of Herbivorous Animal Nutrition, College of Animal Science and Technology, Yangzhou University, Yangzhou, Jiangsu, China; 2 Department of Biology, College of Science, Imam Mohammad Ibn Saud Islamic University (IMSIU), Riyadh, Saudi Arabia; 3 Animal and Fish Production Department, Faculty of Agriculture (Al-Shatby), Alexandria University, Alexandria, Egypt; 4 College of Animal Science and Technology, Yangzhou University, Yangzhou, Jiangsu, China; 5 College of Veterinary Medicine, Yangzhou University, Yangzhou, Jiangsu, China; 6 College of Veterinary Medicine, Albutana University, Rufaa, Sudan

**Keywords:** lactational performance, *Moringa oleifera*, oxidative stress modulation, phytogenic nutraceuticals, reproductive endocrinology, ruminant nutrition

## Abstract

**Introduction:**

*Moringa oleifera* (*M. oleifera*) is highlighted as a diverse-purpose feed in the nutrition of small ruminants. This is a systematic review that compared its effects on goats based on a predetermined PICO model (Population: goats in different physiological phases; Intervention: dietary *M. oleifera* in the forms of leaf meal, extract, or silage; Comparison: no interventions; Outcomes: growth performance, antioxidant status, reproduction, and milk yield and quality).

**Methods:**

The PRISMA guidelines were used to identify the studies from the search included PubMed, Scopus, Web of Science, ScienceDirect, and Google Scholar, and the publication window was January 2015 to December 2025 and screened them with systematic eligibility criteria to minimize selection bias.

**Results and discussion:**

Regular addition of moderate amounts of Moringa supplements had a consistent beneficial effect on nutrient utilization and growth efficiency, and on nitrogen balance, with fibre digestibility improving by around 25%. An intensive antioxidant response was observed, as evidenced by significant increases in enzymatic defenses (e.g., superoxide dismutase upsurge by more than 100% at certain phases) and dramatic decreases in lipid peroxidation (malondialdehyde drops by approximately 70%). Reproductive performance was positively affected by an improvement in endocrine functionality, and progesterone levels during early pregnancy rose up to ~90%.” in supplemented doses, and litter size and offspring viability improved. The benefits of lactational responses were improved milk output and production of milk fat and protein, in addition to a more desirable composition of fatty acids and enhanced oxidative stability of milk. The evidence is that *M. oleifera* is a highly nutritional phytogenic nutraceutical capable of enhancing metabolic efficiency, oxidative balance, reproductive performance, and quality of milk in goats. It produces benefits at moderate dietary inclusion levels, with physiological optimization as opposed to maximal dosing.

## 1 Introduction

Goat production is one of the foundations of food security in the world, especially in developing and climate-prone areas, because of the high degree of adaptation, multi-purpose characteristics, and the compatibility of smallholder production (1). Goats are heavily concentrated in arid, semi-arid, mountainous, and otherwise marginal areas where rainfall is low, soils are poor, and cropping is unreliable (2, 3). In arid areas, Saharan goats have been described as well adapted to harsh climatic conditions and reduced metabolic needs, which supports lactation and survival under water- and feed-limited conditions (4). In semi-arid systems, research from Burkina Faso and Ethiopia shows that goat keeping is embedded in agropastoral and mixed crop-livestock systems, where income, meat, and savings are important reasons for rearing goats (3). In mountainous areas, pasture-based and small-scale livestock systems remain central to rural livelihoods, biodiversity maintenance, and dairy/meat production, with goats frequently highlighted as a major species in mountain livestock farming (5). In these systems, goats rely mainly on native rangelands, browse, shrubs, and crop residues, rather than high-quality cultivated feeds (6). At the domestic level, goats play a very important role in terms of the quality of diets and nutritional security due to the availability of well-balanced foods of animal origin (7). Goats contribute to global food security through both milk and meat, and their value is not confined to dairy systems alone (8). In many countries, goat and sheep meat are important animal-protein sources, and processed sheep/goat meat products remain significant in meat consumption worldwide (9). Goat meat and milk are also great sources of good protein with all the essential amino acids, which are the indispensable building blocks that the human body cannot make and has to be consumed through the diet (10). On the same note, consumption of goat milk has been reported to positively influence the bioavailability of copper, zinc, and selenium in rats (11). The increased bioavailability of such minerals by goat milk is capable of preventing diseases such as anemia especially in areas where iron deficiency is common (12). Essentially, goats form part of household food security, where they provide an easily accessible and nutritious source of food to meet the essential micronutrient deficiencies and offer quality proteins and essential amino acids, especially in resource-limited environments (13). Goat milk makes up a significant portion of human food worldwide with a large proportion of the world population taking goat milk as compared to milk of other livestock species highlighting the significance of goat milk in subsistence and commercial food systems (14).

In socio-economic terms, goat production has a direct effect of reducing poverty, gender equity, and diversification of livelihood. These animals are commonly called a living bank because of the possibility of being sold off when the economy is struggling like in cases of crop failures, medical crises, or climatic disasters (7). The small ruminant industry at the national and regional levels is a major contributor to agricultural GDP as well as one of the quickest expanding livestock subsectors in the low- and middle-income nations (15).

Under the climate change conditions, goats are becoming highly considered as strategic because of their intrinsic adaptability and ability to endure the extreme environmental factors (16). Small ruminants, particularly goats, have a higher ability to survive high temperatures compared to larger ruminants, which survive better with a heat load, as well as achieve better productivity and reproduction (17). Compared to other domestic ruminants, goats do not produce as much methane per head and per pound of body weight, mostly because of their smaller size and relatively efficient feed consumption (18). Large sheep and goat systems also allow the utilization of rangelands with small alternative agricultural utility, which allows the production of food without direct competition with edible crops consumed directly by humans (19). On a systems level Small ruminants can be used to adapt to climate by remaining productive in heat-stressing environments and during feed shortages and their production can be maximized by breed selection of thermotolerant breeds and sustainable feeding methods (20).

**Figure 1:**
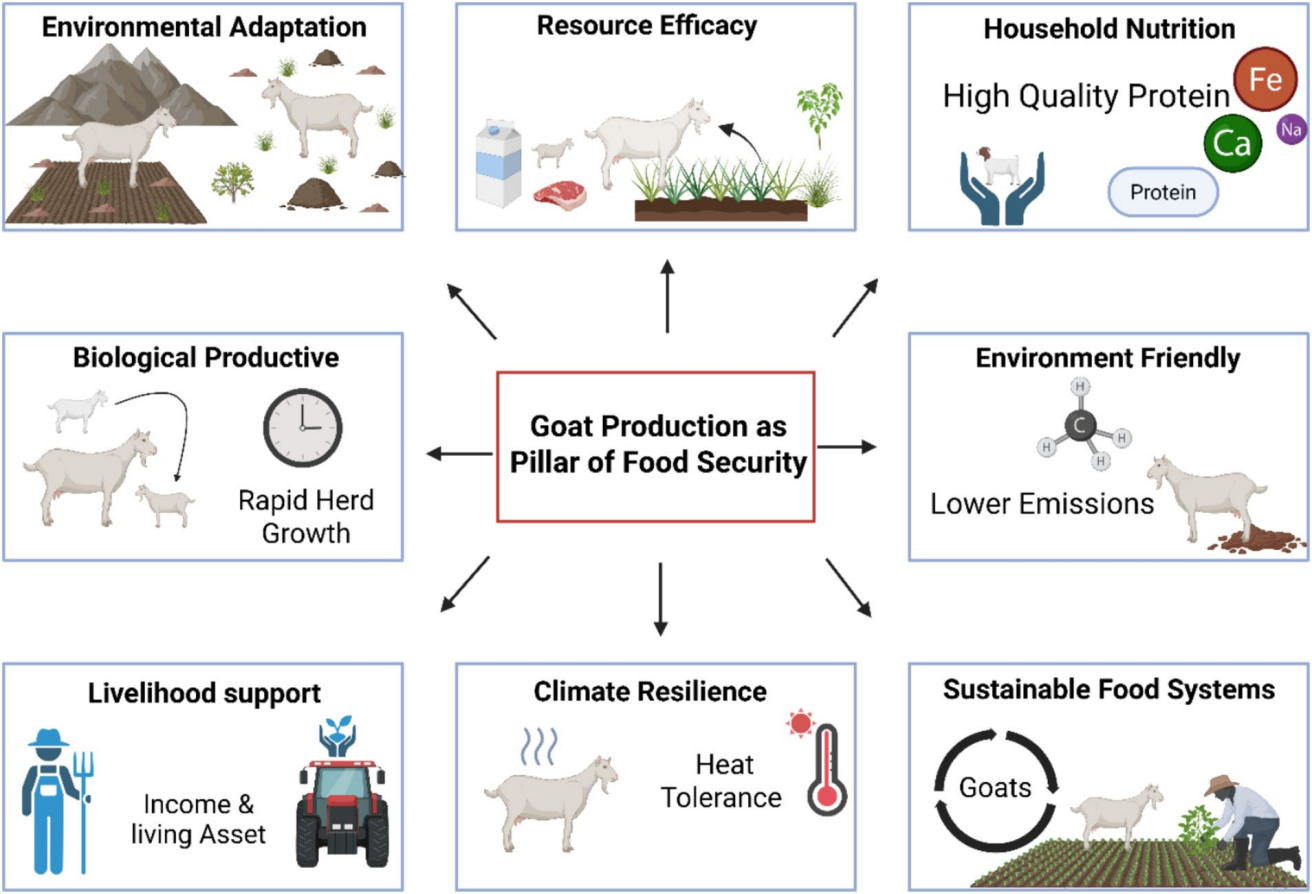
Conceptual framework of the goat production system as a central pillar of global food security. This schematic integrates the biological, environmental, and socio-economic drivers that position caprine production as a resilient livestock model (ex, BioRender).

All these qualities make goat production an essential component of present and future food security plans, especially those in areas experiencing nutritional insecurity, environmental pressure and those without access to traditional livestock feeds (Figure 1). Improvement of goat productivity by addition of nutritional and functional enhanced feeding plans to enhance goat production, thus, is a high-impact avenue to the sustainable livestock production systems in enhancing food availability, quality, and system resilience.

In literature, intensive concentrate feeding of goats enhances growth and production but is evidently linked to rumen fermentation disturbance, lowering fibre-degrading ability, dysbiosis in microbiota, oxidative stress, damage of the barrier and systemic inflammation, which confirm the assertion even outside of the economic scope (20–22). The use of antibiotics, ionophores, chemical antioxidants and other growth promoters has long been applied to increase feed efficiency and rumen fermentation to alter manure metabolism in ruminants (23). Nonetheless, the use of antibiotics in feed at Sub therapeutic levels has been closely associated with antimicrobial resistance (AMR) of animal and human pathogenic organisms, through selection and transmissibility of resistance genes (24). Veterinary antibiotic and synthetic growth promoter residues in meat, milk and eggs increase the risk of allergy, toxicity and resistance in consumers and pollute soil and water through excreta and wastewater (25). Because of them, growth promoters (including ionophores) in feeds were outlawed by the EU in 2006 and numerous jurisdictions are now limiting or winding down the use of growth-promoting and prophylactic antibiotics in feeds (26).

Phytogenic feed additives based on trees, shrubs, herbs, and bioactive fractions have been gaining momentum as an alternative to these limitations and can potentially be used to improve animal performance, along with sustainability and human health goals (27). PFAs (phytogenic feed additives) have been shown to have positive effects in different livestock species, such as non-ruminants and ruminants through, but not limited to, improved growth performance, feed efficiency, gut health, immune modulation, and antioxidant effects (28, 29). PFAs have been revealed to increase weight gain, feed ratio, decrease scouring and mortality, and have a positive effect on intestinal morphology and microbiota composition in broilers and pigs (30–32). Meta-analyses corroborate the idea that PFAs could be successfully used as growth promoters as an alternative to antibiotics as they enhance the production and immune systems with reduced chances of antimicrobial resistance (32). Key sources of phytogenic additives in goat production include *M. oleifera*, a multipurpose tree rich in nutrients and bioactive compounds (33); essential oil-bearing plants such as rosemary and lemongrass (11, 14); tannin-rich species including *Acacia mearnsii*, quebracho, and *Vachellia karroo* (34, 35); and various herbs and spices containing polyphenols, flavonoids, and saponins. Additional sources include purple neem foliage rich in anthocyanins (34), coffee cherry pulp containing diverse phytochemicals (35), and forage legumes and cacti such as *Opuntia ficus indica* (36).

*Moringa oleifera* (*M. oleifera*) is a high economic and nutritional value multipurpose evergreen tree (37). *M. oleifera* has become one of the most popular subjects of research in animal nutrition due to its multifunctionality in the form of high-quality feed and biologically active compounds under both high and marginal conditions as well as arid to semi-arid climates prone to climate change (38). The research indicates that it grows well with low agronomic requirements, which generate quick biomass and high nutrient content of the leaf even in low-fertility or salty soils (39, 40). High levels of crude protein are also a characteristic feature of the *M. oleifera* leaves which is generally about 22 to 30% of dry matter in comparison to the conventional protein supplements that are being incorporated in the ruminant diets (39). Notably, the protein part of *M. oleifera* has a balanced amino acid composition, including significant concentrations of essential amino acids like lysine, methionine, threonine, and valine, which tend to be restrictive in plant-based ruminant diets (41, 42). Besides protein, morphine leaves have high concentrations of various minerals, including calcium, iron, potassium, magnesium, and zinc, that play a role in their overall nutritional value (41). The leaves are also low-fat food but contain positive unsaturated fatty acids and vitamins such as vitamin C and vitamin E (42). *M. oleifera* has a variety of bioactive phytochemicals such as high levels of polyphenols, flavonoids, phenolic acids, condensed tannins, Quercetin, kaempferol, chlorogenic acid, and caffeoylquinic acid are some of the main compounds that make it a functional feed with health-promoting properties (43, 44). These phytochemicals show potent antioxidant, anti-inflammatory, antibacterial, and anticancer properties through the modulation of pathways, such as NF-kappa B, and pro-inflammatory enzyme inhibition (45, 46). Quantitative analyses show significant levels of flavonoids, tannins, terpenoids, and steroids in the leaves compared to other plant parts, supporting their use as both nutritional supplements and natural antioxidants (45).

The polysaccharides in the leaves of *M. oleifera* like MOP-2 and MOP-3 are complex heteropolysaccharides consisting of arabinose, glucose, and galactose, with distinctive glycosidic linkages that make them bioactive (46). These polysaccharides have demonstrated significant immunomodulatory effects by enhancing macrophage activation, increasing pinocytic capacity, stimulating lymphocyte proliferation, and promoting the secretion of immune mediators like reactive oxygen species (ROS), nitric oxide (NO), interleukin-6 (IL-6), and tumor necrosis factor-alpha (TNF-*α*) through upregulation of corresponding mRNA expression in macrophage cells (47). Also, certain arabinogalactans in *M. oleifera* leaves interact with Toll-like receptor 4 TLR4, thereby stimulating the macrophage proliferation, phagocytosis, and cytokine release, indicating a pathway to immune system regulation (48). They also contain anti-inflammatory properties by blocking pro-inflammatory signaling pathways like the NF-kappa B and ensuring the integrity of the intestinal barrier by upregulating tight junction proteins (49). Beyond immune cell activation, Moringa polysaccharides can modulate the gut-associated immune system by influencing gut microbiota composition and promoting anti-inflammatory cytokine secretion like IL-10 (50).

All these distinct convergences of quality protein, balanced amino acids, essential minerals, and a large array of bioactive compounds place the *M. oleifera* in better positions as a functional feed resource as opposed to a traditional forage or protein supplement. The fact that it can effectively contribute to nutrition, antioxidant defense, and immune competence gives it a mechanistic basis for its increasing use in goat production systems to improve productivity, resilience, and sustainability. Despite the growing number of experimental studies investigating *M. oleifera* supplementation in goats, the available evidence remains fragmented and has not yet been consolidated into a comprehensive, goat-specific synthesis. Another critical limitation of the current literature is the lack of integrative evaluation across outcome domains. Many studies focus on isolated endpoints such as growth, milk yield, or antioxidant markers without contextualizing these responses within a broader physiological framework. Therefore, there is a need to critically evaluate and synthesize the existing peer-reviewed evidence on the effects of dietary *M. oleifera* supplementation in goats across different physiological stages, including Metabolism, Reproductive function, and lactation performance.

## 2 Materials and methods

### 2.1 Review design

The proposed systematic review aimed to combine, synthesize, and critically analyze the scientific evidence of the effects of dietary supplementation of *M. oleifera* on productive performance, antioxidant status, immune function, reproductive responses, milk yield, and milk quality in goats. The methodological framework used was systematic, transparent, and reproducible in order to reduce bias and increase the reliability of the conclusions. Reviewing was performed with the principles of the Preferred Reporting Items written under the name Preferred Reporting Items for Systematic Reviews and Meta-Analyses (PRISMA 2020) guidelines, according to which the guidelines give generalized recommendations on the identification of studies, screening, eligibility, and publication of results (51).

A modified PICO framework, adapted for livestock nutrition research (52), was used to define the review question and guide study selection as shown in Table 1.

**Table 1:** PICO framework for the selection of studies.

PICO component	Description applied in this review
Population (P)	Goats (*Capra hircus*), irrespective of breed, age, sex, or physiological stage, including kids, growing goats, lactating does, pregnant animals, and breeding stock.
Intervention (I)	Dietary supplementation with *M. oleifera* in any form, including leaf powder, fresh leaves, dried leaves, aqueous or ethanolic extracts, bioactive fractions (e.g., polysaccharides), or other processed derivatives used as feed additives.
Comparator (C)	Basal diets, conventional feeding systems, or control treatments without inclusion of *M. oleifera* supplementation.
Outcomes (O)	Productive performance indicators (body weight gain, feed intake, feed efficiency), antioxidant status and oxidative stress markers (SOD, CAT, GPx, MDA, TAC, TOS), immune and hematological indices, reproductive and hormonal performance, milk yield and milk composition, rumen fermentation characteristics, rumen microbial ecology, and methane emission parameters.

The review was conducted as a qualitative systematic review employing a narrative synthesis approach rather than a quantitative meta-analysis. This methodological decision was justified by the marked heterogeneity among the available studies. There was a great difference in the form of the used *M. oleifera* which could be in the form of leaf powder, fresh or dried leaves, extracts and processed derivatives and also the technique used in the process and level of inclusion in the diet. Also, there were significant differences between studies in terms of the length of the experiment, physiological stage of animals (growing, lactating, pregnant, or breeding goats), and the basal diets, which affect both metabolic and productive variations. Additional complexity was created by differences in the definitions of the outcomes and analysis methodologies, especially in measuring the antioxidant enzyme, immune biomarkers, and rumen microbial parameters. Collectively, these methodological and biological differences limited the feasibility of statistically pooling data and supported the use of a structured narrative synthesis to critically interpret patterns, consistencies, and divergences across studies. This heterogeneity may lead to the invalidity of pooled effect estimates and has been found to be a significant limitation to meta-analyses in studies on phytogenic feed additives (53). Therefore, the narrative synthesis approach seemed to be more suitable to enable systematic comparison of results, assessment of dose–response curves, and biological mechanism interpretation across studies (54).

### 2.2 Search strategy

To determine the research articles that measured the effectiveness of dietary supplementation of *M. oleifera* in goats a literature search was carried out systematically and in a manner that was comprehensive. PubMed/MEDLINE, Scopus, Web of Sciences, ScienceDirect, and Google scholars were searched to cover as broadly as possible the veterinary, animal nutrition, and ruminant production research, and gray literature. The search dates were between January 2015 and December 2025 to identify the current development on feeding habits and methods of analysis. A combination of search terms based on controlled vocabulary (e.g., MeSH) and free-text keywords with Boolean operators (AND, OR) was used, organized in the form of the PICO framework. Examples of population terms were goat and *Capra hircus*: intervention terms were *M. oleifera*, Moringa leaf powder, and derivatives; outcome terms were growth performance, antioxidant and oxidative stress marker (SOD, CAT, GPx, MDA), immune indices, milk yield and milk composition, reproductive traits and parameters of the fermentation of rumen. One search query was the following representative query: (“goat” OR “*Capra hircus*”) AND (“*M. oleifera*” OR “Moringa leaf powder” OR “Moringa extract”) AND (growth performance) OR (antioxidant) OR (immune function) OR (milk yield) OR (reproduction) OR (rumen fermentation).

### 2.3 Eligibility criteria

Selection of studies was done according to preset criteria in accordance with the PICO model. The included studies were also original *in vivo* studies done on goats (*capra hircus*) of any breed, age, sex, or any physiological stage. Eligible trials evaluated dietary *M. oleifera* supplementation in any form (leaves, leaf powder, extracts, polysaccharides, or derivatives) compared with a basal diet lacking *Moringa*. Research works needed to provide quantitative results associated with productive performance (growth, feed efficiency), antioxidant and oxidative stress biomarkers (e.g., SOD, CAT, GPx, MDA, TAC and TOS), immune or hematological indices, reproductive or hormonal (e.g., milk yield, milk composition) or rumen fermentation, microbial ecology, with clearly described experimental design and treatment levels.

Excluded were those that involved non-goat species, *in vitro* or laboratory-only studies, reviews, abstracts without full data, and trials in which the effects of Moringa could not be disentangled with other plant additives. The other studies that did not include a control group, were not detailed enough methodologically, and did not have quantifiable outcomes, were also excluded as well as the non-English publications and those that were not studied within the period of search.

### 2.4 Study selection

**Figure 2:**
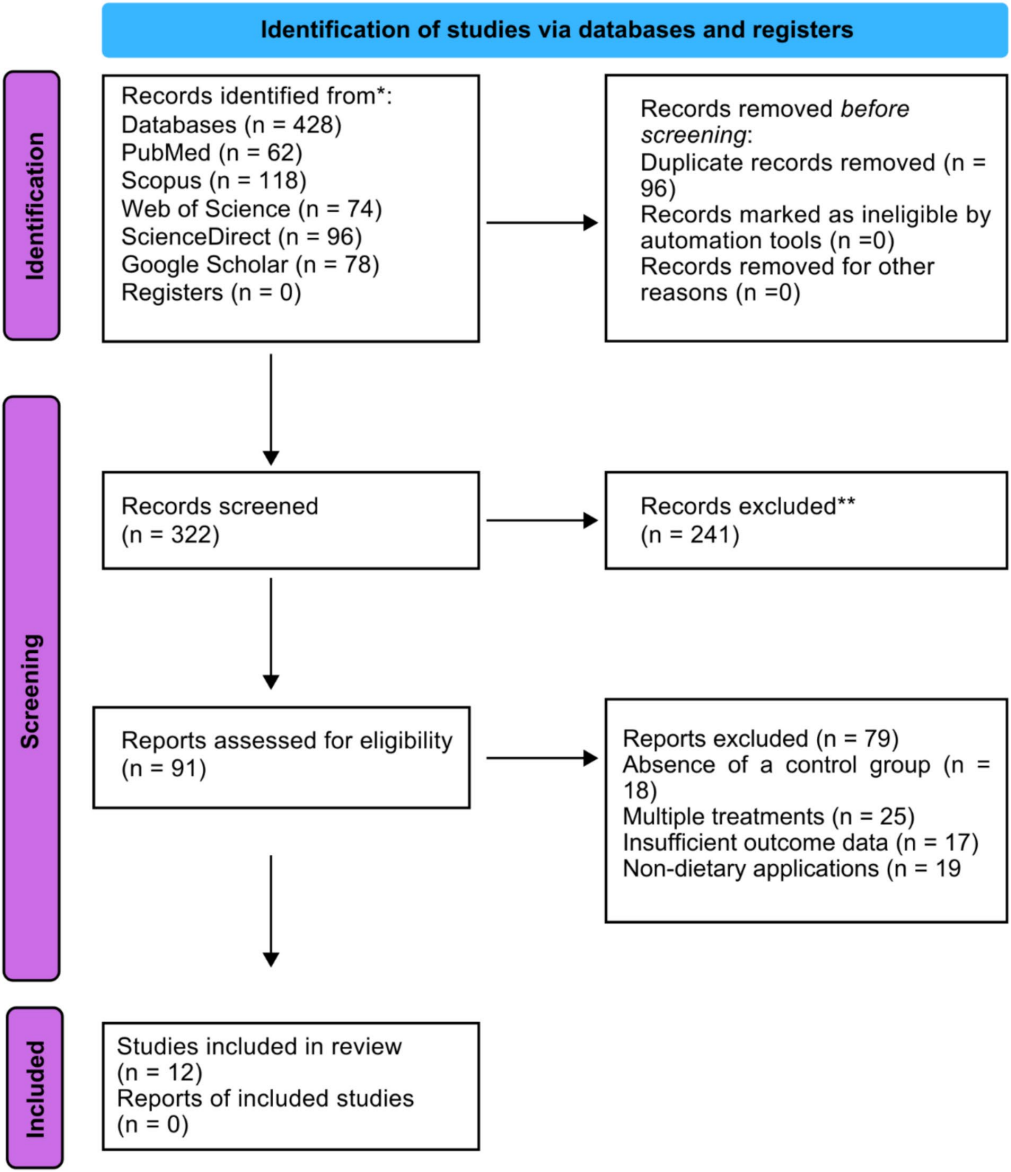
PRISMA 2020 flow chart for new systematic reviews that include only searches of databases (PubMed, Scopus, Web of Science, ScienceDirect, and Google Scholar) and registries.

The study selection process followed PRISMA guidelines and proceeded through sequential phases, shown in Figure 2. The search in the database has produced 428 records (PubMed = 62, Scopus = 118, Web of Science = 74, ScienceDirect = 96 and Google Scholar = 78 other relevant records). On removing 96 duplicates, 332 distinct records were left to screen. At the title and abstract screening, 241 records were left out because they were irrelevant (not goat species, not *Moringa oleifera* interventions, review articles or irrelevant outcomes). This resulted in 91 articles to be subjected to full-text evaluation. The absent reports (*n* = 79) were eliminated in the process of full-text evaluation by preset methodological criteria. The main exclusion criteria were lack of a suitable control group (*n* = 18), the many or confounded effects so that the effect of the use of *M. oleifera* could not be isolated (*n* = 25), inadequate or incomplete quantitative outcome measures (*n* = 17), and non-dietary applications of *M. oleifera* (*n* = 19). Finally, the number of studies relevant according to the criteria of inclusion and exclusion reached 12, which was incorporated in the qualitative synthesis of this systematic review.

### 2.5 Data extraction

Data extraction was performed systematically using a standardized extraction framework to ensure consistency and minimize bias. Outcome data were extracted for all relevant domains, including growth performance and feed utilization, antioxidant and oxidative stress biomarkers, immune and hematological indices, reproductive and hormonal parameters, milk yield and composition, and rumen fermentation characteristics. Where reported, statistical descriptors such as means, standard errors or deviations, *p*-values, and significance levels were recorded to support comparative interpretation.

## 3 Characteristics of included studies

The final evidence ensemble comprises twelve controlled intervention trials (randomized complete-block, Latin-square or completely randomized designs) conducted between 2015 and 2025 across a latitudinal belt of 12–32 ° N, encompassing eight countries (Egypt, Sudan, Nigeria, Pakistan, India, Bangladesh, Israel and China) and four agro-ecological zones (tropical arid, semi-arid, subtropical Mediterranean and humid tropics). Collectively, 471 caprine animals (lactating Damascus, Nubian, Beetal, and Leizhou does; pregnant Nubian females; and growing West-African Dwarf kids) were enrolled, ensuring representation of contrasting physiological states, body weights (3–60 kg), and production systems (small-holder backyard, peri-urban commercial, and intensive research stations). Most studies assessed multiple outcome domains simultaneously, supporting the characterization of *M. oleifera* as a multifunctional feed resource rather than a single-purpose nutrient supplement. Importantly, outcomes were often aligned with physiologically sensitive stages such as early growth, lactation, pregnancy, and reproduction, where dietary modulation is most likely to translate into meaningful biological and productive responses.

There was a high variety of breeds of goats and physiological phases, which made it possible to assess the impact of *M. oleifera* on various goals of production. The studies evaluating milk production, milk quality, antioxidant enrichment of milk, somatic cell count, and reproductive performance mostly employed dairy and dual-purpose breeds, such as Beetal, Nubian, Anglo-Nubian, Baladi, and Saanen x Damascus goats (55–59), The use of growing goats and kids (West African Dwarf goats, local Indian goats and Leizhou goat kids) was mainly aimed at testing average daily gain, feed efficiency, nutrient digestibility, nutrient nitrogen balance, immune development, and rumen development, and these indicators are particularly sensitive to diets with high protein content and bioactive compounds (60–62). Several studies focused on specialized physiological models. Adult cashmere bucks were used to investigate semen quality, reproductive hormone profiles, antioxidant defense, and rumen–testis axis interactions, while pregnant does were studied to assess maternal antioxidant status, progesterone dynamics, and fetal growth trajectories using real-time ultrasonography (63–65). The most widespread form was sun-dried *M. oleifera* leaf powder or leaf meal, which was mainly assessed in terms of feed intake, nutrient digestibility, growth performance, milk yield, milk composition, and reproductive efficiency (55–66). In more mechanistic or functional studies, more processed forms of *M. oleifera* were used. The assessment of milk lipid oxidation, fatty acid profile, and antioxidant transfer to milk was conducted using ensiled whole-plant Moringa, in particular, a-tocopherol enrichment to milk (67). Polyphenol-mediated antioxidant, reproductive, and rumen modulatory effects were isolated by using ethanolic and aqueous leaf extracts, while the purified polysaccharides were proposed in their ability to stimulate immune responses, cytokine patterns, rumen morphology, and microbial community structure in early-weaned kids (55, 61, 64). These variations demonstrate an evolution from whole-feed evaluation toward targeted bioactive intervention.

The availability of inclusion levels was also different, meaning that the form of Moringa and the purpose it was intended to serve differed. Extract based intervention at relatively low doses (mg kg^−1^ body weight or ≤0.3% of diet DM), specific antioxidant, immune, or reproductive pathway, whereas leaf-based interventions were added at moderate or high dietary levels (4–20% of diet DM), some studies substituting up to 75% of dietary protein or forage dry matter to assess nutritional equivalence and productive performance (55, 56). In general, the evidence of positive or neutral results at moderate levels of inclusion was found in most of the studies, and it indicates the practical feasibility of *M. oleifera* supplementation, as well as the need to optimize the dose (Table 2).

**Table 2:** Characteristics of included studies.

No.	Author (year)	Country	Goat breed/physiological stage	Experimental design	Moringa form	Inclusion level/dose	Duration	Main outcomes evaluated	Refs
1.	(Afzal Hussain and Hameed 2021)	Pakistan	Beetal goats; multiparous	Completely randomized design	Sun-dried, ground *Moringa oleifera* leaf powder (MOLP).	0, 4, or 8 g MOLP per doe per day	5 days before estrus synchronization until day 60 of pregnancy (≈ 10 weeks total).	Plasma antioxidant status (non-enzymatic & enzymatic), biochemical indices (protein, sugars, carotenoids, lycopene), progesterone profile, and hydrolytic enzyme activities (protease, esterase, amylase)	(57)
2.	Cohen-Zinder et al. (2025)	Palestine	Saanen × Damascus dairy goats mid-lactation	Completely randomized; *n* = 18; 2 treatments	Ensiled whole-plant *Moringa oleifera*	(25% of forage DM; ~15% of total diet)	6 wk. treatment (+2 wk. covariate)	Milk yield & composition somatic-cell count *α*-tocopherol MDA phospholipids *ω*-3 FA n-6/n-3 ratio	(67)
3.	Leitanthem et al. (2023)	India	Local male goat kids 3–4 mo under berseem-based diets	Randomised block; *n* = 24; 4 groups	Sun-dried *Moringa oleifera* leaf powder	0 4 6 4% of diet DM (10% or 20% of concentrate)	180 d feeding +7 d metabolic trial	Nutrient digestibility N-balance ADG FCR enteric CH₄ blood biochemistry & antioxidant enzymes immunity	(55)
4.	Liang et al. (2023)	China	Adult cashmere bucks	Completely randomised; *n* = 21; 3 groups	Ethanolic extract (MOLE) or dry leaf powder (MOLP)	40 mg kg^−1^ body weight, given orally once daily	56 days	Semen quality (volume, concentration, motility, viability, abnormality), blood antioxidant status (SOD, CAT, GSH-Px, T-AOC, MDA), reproductive hormones (GnRH, testosterone), rumen microbiota composition & rumen metabolome (PUFA, steroid hormones)	(60)
5.	(J. Liu et al. 2024)	China	Leizhou male goat kids	Completely randomized design	Commercial *Moringa oleifera* leaf polysaccharide powder	0.15% or 0.3% of daily dry-matter intake, mixed into milk replacer	54 days (from 7 to 60 days of age)	BW, ADG, feed intake, serum biochemistry and antioxidant status, immunoglobulins and cytokines, rumen morphology, rumen fermentation parameters, rumen microbial community.	(61)
6.	Afzal et al. (2022)	Pakistan	Multiparous Beetal dairy goats	Randomized complete block design	Sun-dried, ground *Moringa oleifera* leaf powder (MOLP)	1.6% or 3.2% of total diet DM	60 days	Plasma & milk biochemistry milk yield & composition BW change reproductive performance	(63)
7.	Farrag (2024)	Egypt	Baladi goats/Lactating and breeding	Randomized design	Sun-dried, ground *Moringa oleifera* leaves mixed into the concentrate	10% or 20% of total diet dry matter	120 days	Reproductive performance, hematology, milk yield and composition	(59)
8.	Okpara et al. (2025)	Nigeria	West African Dwarf goats/Growing	Completely randomized design	Sun-dried *Moringa oleifera* leaves	0, 5, 10, 15% or 20% of total diet DM	84,120 days	Growth performance (ADG, FCR), feed intake, nutrient digestibility (DM, CP, EE, ash, NDF, ADF), blood biochemical indices	(56)
9.	Kholif et al. (2015)	Egypt	lactating Anglo-Nubian goats	4 × 4 Latin square	Sun-dried *Moringa oleifera* leaf meal (MOLM)	25, 50 or 75% of protein source	84 d	Feed intake, rumen fermentation, milk yield and composition, milk fatty-acid profile	(69)
10.	Kholif et al. (2018)	Egypt	Lactating Nubian goats	Quadruplicated 4 × 4 Latin squareSun-dried *Moringa oleifera* leaf meal (MOLM)	aqueous Moringa-leaf extract (MLE)	0, 10, 20, 30 g MLE per kg total diet	84 d	feed intake, nutrient digestibility, ruminal fermentation	(64)
11.	Kholif et al. (2017)	Egypt	Lactating Nubian goats	Quadruplicated 4 × 4 Latin square	Sun-dried *Moringa oleifera* leaf powder (MOLP)	25, 50 or 75% of forage DM	88 days	Feed intake and digestibility, milk yield (+6%), milk energy, milk fatty-acid profile (CLA ↑ 17–23%), protein, lactose, SNF, TS	(62)
12.	Husien et al. (2025)	Sudan	Pregnant Nubian does	Completely randomised	Sun-dried *Moringa oleifera* leaf powder (MOL)	0, 20, 40 or 60% of total diet DM (≈ 0, 120, 240 or 360 g MOL/d)	Full gestation period (~135 days)	Real-time fetal biometry: crown-rump length (CRL), biparietal diameter (BPD), femur length (FL); correlations with gestational age	(58)

## 4 Results and discussion

### 4.1 Growth performance and nutrient utilization

The economic metrics of goat production are growth performance and nutrient utilization, which directly affect the feed ratio, time-to-market, and profitability as a whole (65). Traditional protein supplements like fishmeal and soybean meal are impractical in regions with low levels of economic resources. It is therefore important to find some local and climate-tolerant forages that will be efficient in keeping the positive nitrogen balance and promote the growth of lean muscle tissue in the livestock (66). *M. oleifera* is a potential feedstock here, with crude protein levels (220–280 g/kg DM) comparable to alfalfa and insignificant tannin levels, but significant bioactive polyphenols. Moderate inclusion of *Moringa oleifera* in the diet, including levels up to 10–20% of concentrate mix or total dietary DM in some goat studies, has been associated with improved digestibility, feed efficiency, and average daily gain, although the response is dose-dependent and may decline at the highest inclusion level (55, 68).

Leitanthem et al. (53) showed that the partial replacement of concentrate by *M. oleifera* leaf meal had a significant positive effect on growth performance and nitrogen utilization in growing goat kids during a 180-day feeding trial. The initial body weight did not show a significant difference among treatments (*p* = 0.981), which ensured the baseline comparison conditions. This study collectively reported 14–15% greater final body weight in goats receiving 4–6% *Moringa* leaf meal. Average daily gain increased by 27–28% (101.01 vs. 79.22 g/day; *p* < 0.001), while feed conversion efficiency improved by ~18% (13.41 vs. 11.38; *p* < 0.003). Nitrogen balance rose by ~25% (2.87 vs. 2.29 g/day, *p* = 0.01), indicating improved protein retention without increased excretory losses.

Okpara et al. (56) tested graded levels of inclusion of the *M. oleifera* leaves (0–20% of diet DM) as a supplement to a basal diet of Guinea grass-oil palm fronds in growing West African Dwarf (WAD) goats. At 10% inclusion, goats achieved the highest ADG (34.42 g/day) and improved feed conversion ratio (FCR = 18.28 vs. 23.40 in controls; *p* < 0.05), while crude protein digestibility rose from 60.0 to 65.4%. However, excessive inclusion (20%) diminished fibre digestibility (NDF digestibility from 60.2 to 54.9%), highlighting the biphasic effect of phenolic bioactives that can enhance nitrogen efficiency at moderate levels but suppress fibrolytic microbes at higher doses. Such responses align with other findings where plant secondary compounds exert hormetic effects on rumen ecology.

Kholif et al. (69) measured the impact of substituting sesame meal with *M. oleifera* leaf meal (MLM) on feed intake, nutrient digestibility, and dietary energy consumption among lactating Anglo-Nubian goats in a quadruplicated 4 × 4 Latin square study. It is reported that replacing sesame meal with Moringa leaf meal (up to 15%) substantially increased dry matter intake (DMI) and the digestibility of DM, organic matter (OM), and fibre fractions (NDF and ADF; *p* < 0.0001). Although crude protein digestibility declined modestly (~6 percentage units), this likely reflects tannin protein complexation in the rumen, a mechanism often exploited to protect dietary protein from premature ruminal breakdown and enhance post-ruminal amino acid availability. The net energy of lactation also increased as rumen fermentation shifted toward higher fibre and carbohydrate utilization.

Aqueous *Moringa oleifera* leaf extract supplementation in lactating Nubian goats, as reported by Kholif et al. (64) enhanced nutrient intake and utilization without altering body weight dynamics during early lactation. Initial and final body weights, as well as daily weight change, were not significantly affected (*p* > 0.05), confirming preferential energy partitioning toward milk production. Dry matter intake increased by approximately 10% (751 to 828 g/day), while crude protein intake rose by about 9–10% (130 to 143 g/day) compared with the control (*p* < 0.001). Dry matter digestibility improved by roughly 8–9% (541 to 588 g/kg), and neutral detergent fibre digestibility increased by about 7–8% (552 to 595 g/kg). Significant improvements were also observed in organic matter, acid detergent fibre, hemicellulose, and cellulose digestibility, whereas crude protein digestibility remained unaffected. These enhancements are translated into improved dietary energy utilization. Net energy of lactation increased by approximately 6% (1.13 to 1.20 MJ/kg DM), alongside parallel increases in total digestible nutrients, digestible energy, and metabolizable energy (*p* < 0.001).

**Figure 3:**
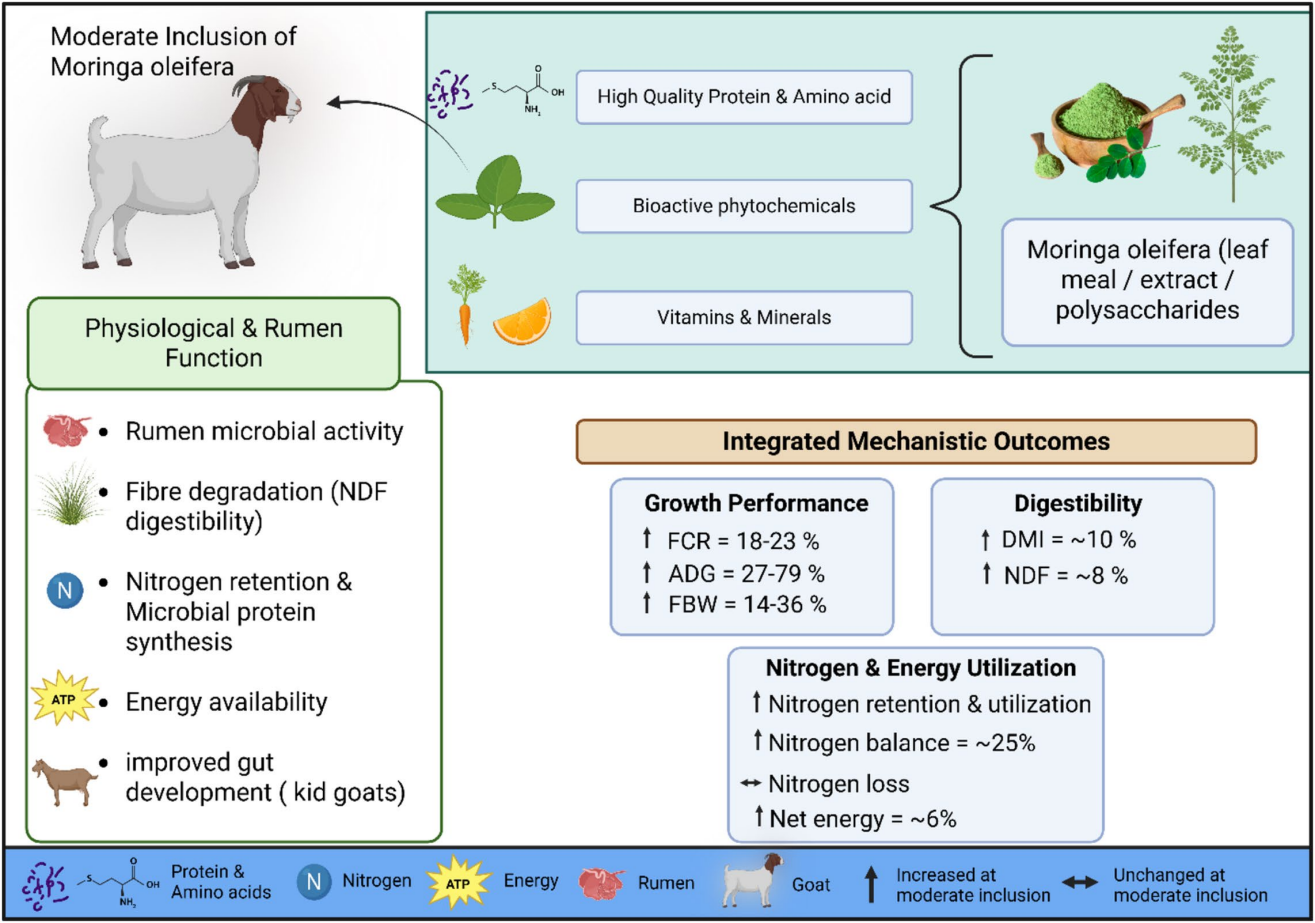
Moderate inclusion of *M. oleifera* in rumen functions and physiological performances of goats: mechanical effects. This flow chart shows the physiological mechanisms that the dietary intake of *M. oleifera* (leaf meal, extract, or polysaccharides) can modify, since this dietary ingredient is a high-quality protein, essential amino acids, and bioactive phytochemicals. These ingredients improve the growth of microbes and fiber breakage in the rumen, resulting in the maximization of nitrogen retention as well as energy. The integrated mechanistic outcomes demonstrate substantial improvements in growth performance, including Feed Conversion Ratio (FCR: 18–23%), Average Daily Gain (ADG: 27–79%), and Final Body Weight (FBW: 14–36%). Additionally, increased Dry Matter Intake (DMI: ~10%) and Neutral Detergent Fiber (NDF) digestibility (~8%) contribute to a 25% improvement in nitrogen balance and higher net energy yields, supporting enhanced gut development in kid goats. (Biorender).

In early-weaned Leizhou goat kids, supplementation with *Moringa oleifera* polysaccharides (MOP), as reported by Liu et al. (61), markedly improved post-weaning growth performance without affecting milk replacer intake. Initial body weight did not differ among groups (*p* = 0.976); however, final body weight increased by approximately 29–36% (6.95–7.36 vs. 5.40 kg; *p* = 0.009) in the supplemented groups compared with the control. Average daily gain increased by about 63–79% (73.45–80.54 vs. 44.96 g/day; *p* = 0.020). Milk replacer intake remained unchanged (*p* = 0.938), indicating that growth enhancement was not due to higher liquid feed consumption. Instead, starter feed and green hay intake significantly increased (*p* = 0.032 and *p* = 0.003), with starter intake rising by more than 80% relative to the control. Collectively, these findings suggest that MOP supplementation accelerates rumen functional development and solid-feed adaptation, likely through prebiotic modulation of gut microbiota and mucosal maturation, thereby improving feed utilization efficiency and growth performance in early-weaned goats (Figure 3).

Concluding all studies, at a mechanistic level, the improvements in growth performance and nutrient utilization associated with *M. oleifera* supplementation arise from three closely interconnected processes (Figure 2). First, enhanced nitrogen retention and utilization occur as a result of the provision of fermentable protein and partial rumen-sparing effects, reflected in increases in nitrogen balance of approximately 25% under optimal inclusion levels. Such improvements are consistent with the high rumen-degradable protein and balanced amino acid profile of *Moringa* leaves, which enhances microbial protein synthesis and growth efficiency in small ruminants (70). In cross-species examinations, we can see these improvements in previous studies. For instance, dietary inclusion of *Moringa* leaf meal significantly enhanced dry matter intake, daily weight gain, and feed conversion efficiency in buffalo calves by improving rumen fermentation and metabolic hormone profiles such as insulin-like growth factor-I (IGF-I) (71). Second, fibre and carbohydrate digestibility are improved, with neutral detergent fibre digestibility increasing by up to about 8% at moderate supplementation, likely due to favorable shifts in rumen microbial fermentation patterns and greater production of volatile fatty acids that support energy metabolism. Evidenced by *in vitro* addition of *M. oleifera* leaves increased total gas production and improved in vitro digestibility of dry matter and organic matter (indicative of enhanced fermentation) in mixed ruminant substrates at 10–20% inclusion (72). On sheep, *M. oleifera* supplementation (2.5% of concentrate) with or without live yeast improved the digestibility of dry matter and organic matter, and increased total digestible nutrients and digestible energy (73). Similarly, Broiler chickens receiving Moringa leaf extracts or powder exhibited higher ADG, better feed conversion ratios, and improved health parameters compared to controls (74, 75). Third, overall energy intake and efficiency are elevated, as shown by increases in dry matter intake of roughly 10% and net energy values of about 6%, promoting more effective energy partitioning toward productive tissue gain. For instance, replacing 20% of concentrate with Moringa leaf silage improved ADG and feed efficiency in growing goat kids by enhancing nutrient digestibility and rumen microbial populations (75). Similarly, Sanwo et al. investigated that feeding *M. oleifera* leaf meal to West African Dwarf sheep improved weight gain and affected blood chemistry and carcass traits (76). In other species, we can observe similar effects. In rabbits, supplementation with 10–15 g/kg *Moringa leaf* powder increased final live weight and ADG by improving antioxidant capacity and nutrient utilization (77). In pigs, inclusion of dehydrated Moringa leaves up to 7% enhanced weight gain without negatively affecting digestibility (78). Nevertheless, when phytochemical concentrations exceed optimal thresholds, fibrolytic activity may be impaired, with reductions in fibre digestibility of up to around 9%, underscoring the importance of maintaining moderate supplementation levels, generally in the range of 4–15% of dietary dry matter or equivalent extract doses The summary of Growth performance and nutrient utilization outcomes in goats supplemented with *M. oleifera* is given (Table 3).

**Table 3:** Summary of Growth Performance and Nutrient Utilization Outcomes in Goats Supplemented with *M. oleifera.*

Study	From of *Moringa oleifera*	Inclusion level/treatment	Growth performance outcomes	Nutrient utilization outcomes	Key statistical evidence	Refs
Leitanthem et al. (2023)	Moringa leaf meal (ML)	4–6% of diet DM replacing concentrate	Final BW and ADG increased in GII and GIII vs. control; the highest ADG in GII	Improved N intake and N balance; FCR similar among groups	Final BW (*p* = 0.005); ADG (*p* < 0.001); N balance (*p* = 0.01)	(55)
Okpara et al. (2025)	Moringa leaf meal	Partial replacement of the conventional protein source	Increased BW gain and feed efficiency at moderate inclusion; reduced performance at high levels	Improved DM and fibre digestibility at low–moderate levels; CP digestibility reduced at high inclusion	Digestibility improvements (*p* < 0.05); CP digestibility decline at high dose	(56)
Kholif et al. (2015)	Moringa leaf meal	Incremental dietary inclusion	No change in BW; indirect support for production via improved feed use	Increased DM, OM, NDF, ADF, cellulose digestibility; reduced CP digestibility	Fibre digestibility (*p* ≤ 0.0157); CP digestibility (*p* < 0.05)	(69)
Kholif et al. (2018)	Moringa leaf extract	0, 10, 20, 40 mL/day	No differences in BW change among treatments	Increased nutrient intake and digestibility; higher TDN, DE, ME, NEL with ME20–ME40	Intake & digestibility (*p* < 0.001); energy values (*p* < 0.001)	(64)
Liu et al. (2024)	*Moringa Oleifera* polysaccharides.(MOP)	0.15 and 0.3% of DMI in milk replacer	Final BW and ADG significantly higher in LOW and HIG groups vs. control	Increased starter and hay intake, indicating enhanced nutrient utilization	Final BW (P = 0.009); ADG (*p* = 0.020); starter intake (*p* = 0.032)	(61)

BW, body weight; ADG, average daily gain; DM, dry matter; CP, crude protein; NDF, neutral detergent fibre; ADF, acid detergent fibre; FCR, feed conversion ratio; TDN, total digestible nutrients; DE, digestible energy; ME, metabolizable energy; NEL, net energy of lactation.

### 4.2 Antioxidant status and oxidative stress mitigation

The production of reactive oxygen species is increased during such periods in high-producing goats as late pregnancy and early lactation, and when the species surpasses the antioxidant capacities of the animal, there is an imbalance that results in oxidative stress (79). The periparturient period is characterized by high levels of lipolysis and the release of free fatty acids, which are oxidized in the mitochondria; these excessive loads augment the activity of the electron transport and result in the amplified generation of reactive oxygen species, including superoxide anions, hydrogen peroxide, and hydroxyl radicals (80, 81). High ambient temperatures increase heat stress, cortisol, and production of reactive oxygen species, with a decrease in the activity of important antioxidant enzymes such as superoxide dismutase (SOD) and catalase in the case of tropical goats. This situation stimulates lipid peroxidation, suppresses immunity, and leads to decreases in milk production during long-term exposure to heat (82, 83). Dietary antioxidants help nutrients, including high levels of vitamin C, carotenoids, vitamin E, and polyphenols such as quercetin, kaempferol, and caffeic acid that can scavenge reactive oxygen species, chelate pro-oxidant metal ions, and enhance the activity of endogenous antioxidant (84, 85). These components are important because they have a multimodal antioxidant cascade. Vitamin C is a water-soluble antioxidant, which reacts with water-soluble reactive oxygen species, superoxide and hydroxyl radical, as well as the ability to regenerate oxidized vitamin E, forming a synergistic loop of antioxidants (85). Quercetin and kaempferol can chelate redox-active iron (Fe2+) and copper, to suppress the hydroxyl radical generation through Fenton reactions and consequently, lipid peroxidation. They also inhibit NF-kB signaling, reduce the expression of pro-inflammatory cytokines and the related production of ROS (86, 87). Thus, the question of the impact of the *M. oleifera* supplementation on the Antioxidant status and mitigation of oxidative stress in goat production remains uninvestigated.

The effects of *M. oleifera* leaf powder (MOLP) supplementation on oxidative balance in pregnant Beetal goats were evaluated (57). These effects were found to be consistent and dose-dependent in improving antioxidant capacity and reducing oxidative stress biomarkers throughout gestation. This study showed that inclusion of MOLP at 3.2% DM produced a substantial shift toward a reduced oxidative state by late gestation. Total antioxidant capacity (TAC) increased by ~92% (2.29 vs. 1.19 μM/mL; *p* = 0.004), while total oxidant status (TOS) declined by ~8.6% (1,376 vs. 1,505 μM/mL; *p* < 0.001). Lipid peroxidation was dramatically suppressed, with plasma MDA decreasing by ~77% (1.95 vs. 8.34 μM/mL; *p* = 0.038). These changes coincided with marked increases in circulating antioxidants, including vitamin C (+6.5%), flavonoids (+11%), and phenolics, indicating that MOLP directly enriched the systemic antioxidant pool while enhancing endogenous defenses. Enzymatic activity showed even stronger responses: SOD increased by ~133% (34.8 vs. 14.9 U/mL; *p* = 0.002), POD by ~18%, and CAT by ~26%, demonstrating a coordinated enhancement of superoxide dismutation and hydrogen peroxide detoxification capacity.

Leitanthem et al. (68) provided robust evidence that dietary inclusion of *M. oleifera* leaf powder (ML) enhances systemic antioxidant capacity and mitigates oxidative stress in growing goat kids. The activities of the major endogenous antioxidant enzymes, including glutathione peroxidase (GPx), catalase (CAT), and superoxide dismutase (SOD), were relatively high at graded levels of ML inclusion as compared to the control diet without ML. The GPx activity of 14.17 ± 0.18 mmol/g Hb NADPH oxidized has enhanced in goats fed with 6% ML to an activity of 15.03 ± 0.27 mmol/g Hb (*p* < 0.001). On the same note, the CAT activity increased from 54.17 ± 0.18 to 55.03 ± 0.27 μmol H₂O₂ consumed/min/g Hb (p < 0.001), and SOD increased from 91.69 ± 0.29 to 93.40 ± 0.34 U/mg Hb (*p* = 0.003). Notably, the improvements did not accompany increasing levels of hepatic enzyme markers since the plasma AST, ALT, and ALP activities were not significantly different across the dietary treatments (*p* > 0.47). This reveals that the increase of antioxidant defenses caused by ML was not linked to the stress or metabolic impairment of hepatic cells. Taken together, Comparable patterns were presented in the study by Leitanthem et al. (68) where dietary ML increased the GPx activity by approximately 6% and CAT by approximately 1.6 and SOD by approximately 1.9% (all *p* ≤ 0.003) without an increase in the hepatic enzyme markers (AST, ALT, ALP), which proved that the antioxidant upregulation was maintained without metabolic stress.

Afzal et al. (63) reported, Supplementation with 3.5% *Moringa oleifera* leaf powder (MOLP) produced some of the most pronounced improvements in systemic antioxidant status during early lactation. Superoxide dismutase activity increased by approximately 138% (42.76 vs. 17.97 U/mL; *p* = 0.001), catalase by about 26%, and peroxidase by roughly 18%. Total antioxidant capacity rose by approximately 41%, whereas total oxidant status declined by about 8–9%. Notably, plasma malondialdehyde decreased by nearly 65% (2.74 vs. 7.94 μM/mL; *p* = 0.013), indicating strong suppression of lipid peroxidation.

**Figure 4:**
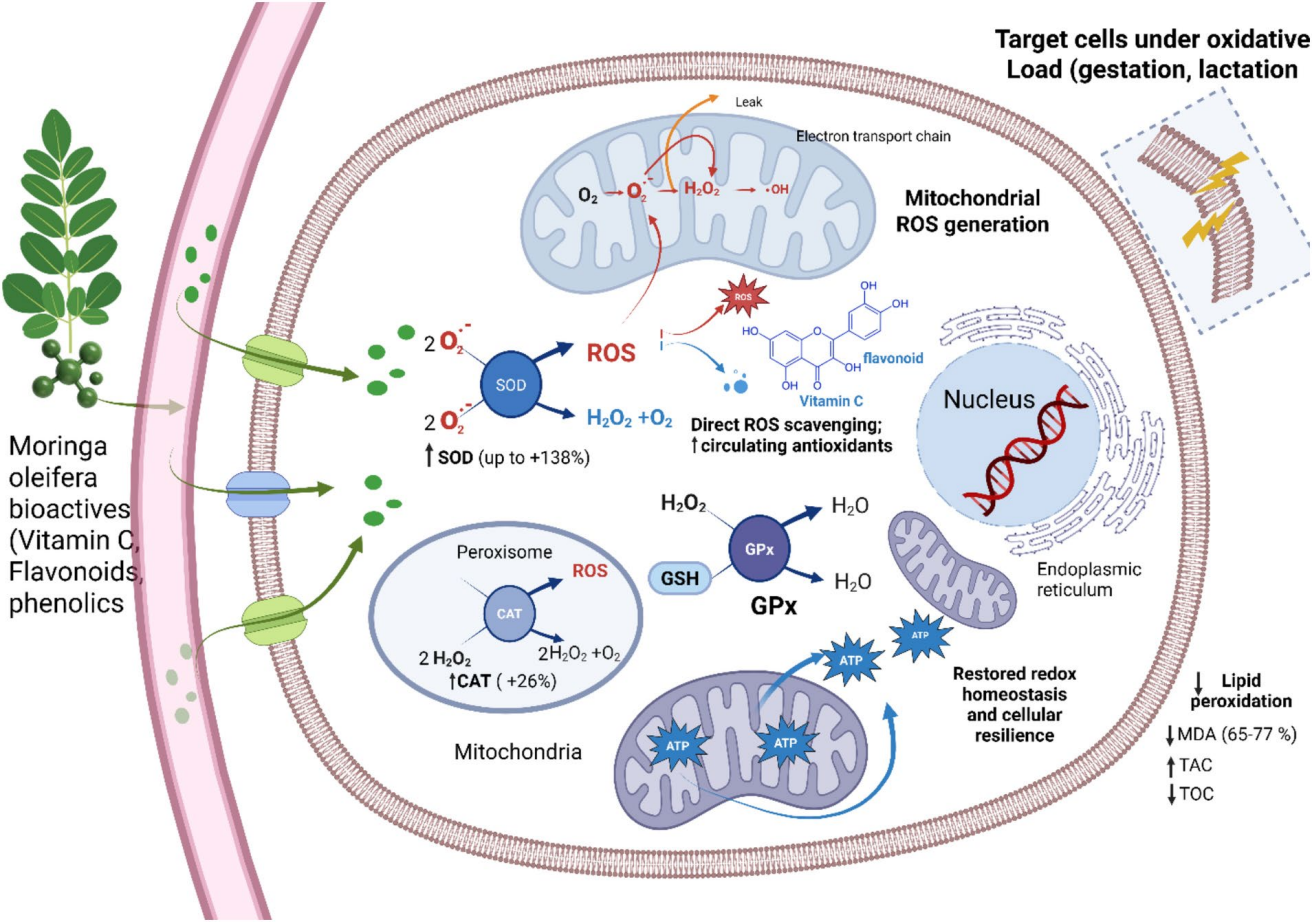
*Moringa oleifera* supplementation mitigates oxidative stress in goats by reinforcing intracellular redox regulation, particularly during gestation and lactation. Bioactive compounds (vitamin C, flavonoids, phenolics) enter target cells and enhance enzymatic (SOD, CAT, GPx, POD) and non-enzymatic antioxidant defenses. Mitochondrial electron leakage generates ROS (O_2_•-, H_2_O_2_, •OH), which are efficiently detoxified through coordinated enzymatic conversion and direct radical scavenging, increasing total antioxidant capacity (TAC). Consequently, lipid peroxidation (MDA) and total oxidant status (TOS) decline, preserving membrane integrity, mitochondrial function, and DNA stability. These adaptations restore cellular redox homeostasis and improve metabolic resilience without inducing hepatic stress markers (Biorender).

*M. oleifera* supplementation was always able to increase redox homeostasis by different synchronized biological processes (Figure 4). First, it increased the non-enzymatic antioxidant reserves as indicated by an increase in the circulation of flavonoids (≈10–11%), vitamin C (≈6–7%), and total phenolics, and thus enhanced direct free-radical scavenging capacity. Antioxidant profiling of the Moringa leaf extracts shows high levels of total phenolics and flavonoids, which are associated with good antioxidant activity in DPPH and ABTS radical scavenging activities, which are consistent with their ability to scavenge free radicals *in vivo* (87). In the same manner, according to the study by Gama M. El-sherbiny et al. Moringa leaves contain phenolic acids and flavonoids (quercetin and kaempferol) known to provide antioxidant activity in biochemical tests that determine radical scavenging and redox potential (88). Cross-species examination also testifies to it. As an example, dairy cow supplementation with Moringa leaf flavonoid increased the total antioxidant potential and activity of antioxidant enzymes, showing a systemic increase in the antioxidant reserves (89). *Moringa oleifera* meal-supplemented sows exhibited increased antioxidant enzyme activities and decreased malondialdehyde, indicating that antioxidant reserves were increased and the redox situation became better in both sows and piglets (90).

Second, it provoked endogenous enzyme defense mechanisms, and significant increases in superoxide dismutase (up to ≈138%), glutathione peroxidase (≈6%), and catalase (≈26%) enhanced the detoxification of superoxide anions and hydrogen peroxide in cells. A vitro experiment examined the ability of *M. oleifera* leaf extract to elevate activities of superoxide dismutase (SOD), catalase (CAT), and glutathione peroxidase (GPx) in C2C12 myotubes, which are in the oxidative stress environment (91). Extracts of *M. oleifera* improved redox homeostasis in C2C12 skeletal muscle cells through the upregulation of Nrf2 and downstream antioxidant enzymes, such as SOD, CAT, and GPx, in a mechanistic cell model (92). Similarly, Dietary Moringa leaf powder improved the activity of antioxidant enzymes (SOD, GPx, and CAT) in lactating buffalo serum, which was an indication of the systemic stimulation of endogenous enzyme defenses (93). Flavonoids from *M. oleifera* leaves significantly elevated SOD, CAT, and GPx activities in bovine mammary epithelial cells under oxidative challenge, supporting enhanced detoxification capacity in a livestock cellular model (94). Third, such antioxidant improvements were also reflected in practical inhibition of oxidative damage as assessed by significant decreases in malondialdehyde (≈65–77%) and total oxidant status (≈8–9%) and significant increases in total antioxidant capacity (up to ≈92%), which all point to a systemic change to a more reduced and protective oxidative state. The aqueous *M. oleifera* extracts enhanced glutathione, and decreased MDA at concentration-dependent ways in the *in vitro* antioxidant activities, which validates its role in reducing lipid peroxidation and oxidative harm (95). Dietary *Moringa oleifera* supplementation has been associated with improved antioxidant status, including higher total antioxidant capacity and antioxidant enzyme activity, together with lower malondialdehyde (MDA), indicating enhanced oxidative stability (89). The progressive strengthening of these responses during late gestation and early lactation highlights the capacity of *M. oleifera* to counteract reproduction-associated oxidative stress. Notably, these benefits occurred without elevations in hepatic enzyme markers, suggesting that the antioxidant modulation represented physiological adaptation rather than stress-induced pathology. Integrated outcomes are given in Table 4.

**Table 4:** Effects of *M. oleifera* on antioxidant status and oxidative stress mitigation in goats.

Study	Animal category	Moringa form and inclusion	Key antioxidant and oxidative stress outcomes (mean ± SEM)	Enzymatic antioxidant response (mean ± SEM)	Critical interpretation (direction of effect)	References
(Afzal Hussain and Hameed 2021)	Beetal does (multiparous)	MOLP, 4 g and 8 g/day	TF: 311.99 ± 0.51 μg/mL; TPC: 5,948 ± 0.41 μM/mL; Vit C: 871.75 ± 0.47 μg/mL; MDA: 1.95 ± 0.41 μM/mL; TOS: 1,376.25 ± 0.47 μM/mL	SOD: 34.80 ± 0.42 U/mg Hb; CAT: 51.00 ± 0.41 U/mL; POD: 290.48 ± 1.88 U/mL	↑↑ Antioxidant capacity, ↓↓ lipid peroxidation, ↓↓ oxidative load; strongest response under gestational stress	(57)
Leitanthem et al. (2023)	Growing goat kids	Moringa leaf powder, 4–6% DM	Oxidative stress markers not directly measured; plasma biochemical stability maintained	GPx: 15.03 ± 0.27 μmol NADPH oxidized/g Hb; CAT: 55.03 ± 0.27 μmol H₂O₂/min/g Hb; SOD: 93.40 ± 0.34 U/mg Hb	↑ Basal antioxidant enzyme activity, ↔ oxidative stress status (preventive effect rather than corrective)	(55)
Afzal et al. (2022)	Beetal goats	MOLP, 5 g and 8.75 g/day	TF: 291.44 ± 0.63 μg/mL; TPC: 5,884 ± 1.08 μM/mL; Vit C: 838.69 ± 1.25 μg/mL; MDA: 2.74 ± 0.29 μM/mL; TOS: 1,459.75 ± 0.48 μM/mL	SOD: 42.76 ± 0.36 U/mL; CAT: 63.39 ± 0.86 U/mL; POD: 268.11 ± 0.72 U/mL	↑↑ Antioxidant defense, ↓↓ oxidative stress around parturition, ↑ redox resilience during lactation onset	(63)

### 4.3 Reproductive and hormonal performance

Reproductive efficiency is one of the determinants of the sustainability and profitability of herds, which is very susceptible to disturbances caused by lack of proper nutrition, oxidative stress, and metabolic imbalance that disrupt reproductive processes and fertility (96, 97). It seems to be especially susceptible during the periparturient and early-pregnancy stages: negative energy balance inhibits gonadotropin-releasing hormone (GnRH) pulpability, decreases the size of luteinizing hormone (LH) surge, and inhibits corpus luteum production of progesterone, resulting in delayed conception, embryonic death, and protracted kidding periods (98, 99). In ruminants, negative energy balance is associated with lower glucose, insulin, IGF-1, and leptin concentrations, together with slower ovarian follicular growth, showing that reduced metabolic fuel directly compromises ovarian activity (98, 99). This is added to by oxidative stress which destroys ovarian granulosa cells and oocyte mitochondrial membrane, reducing fertilization competence and conceptus survivability (100). The existence of dietary antioxidants has consequently been suggested to protect reproductive tissues against ROS injury, balance the effectiveness of the hypothalamic–pituitary-gonadal (HPG) axis, and promote the development of the placenta (101). *M. oleifera* is best suited to this purpose: the leaves provide not only antioxidant vitamins (A, C, E) (102) and polyphenols, but also essential amino acids (arginine, methionine) that serve as precursors for hormone synthesis, and minerals (zinc, selenium) that act as enzymatic cofactors in steroidogenesis (103). The combination of these nutrients in MOLM, in theory, forms an environment of reproductive-supportive metabolism, which increases conception rates, reduces kidding intervals, and fetus development.

Afzal Hussain and Hameed et al. (57) investigated the influence of *M. oleifera* leaf powder (MOLP) supplementation on reproductive endocrinology in multiparous Beetal goats during early pregnancy. MOLP treatment in goats showed a definite dose-dependent increase in the plasma concentrations of progesterone during the experiment. During the initiation of gestation (day 0), the plasma progesterone levels were considerably higher in goats fed with 3.2 g MOLP per day (0.78 ng/mL) than in the control group (0.65 ng/mL; *p* ≤ 0.05). This deviation continued to increase with gestation. After 30 days, the concentrations of progesterone increased to 4.55 ng/mL in the higher MOLP group, which was higher than those of the control (3.31 ng/mL) and lower supplementation group (3.78 ng/mL; *p* ≤ 0.05). The same trend continued until day 45 of pregnancy, with 3.2% MOLP group recording the highest progesterone concentration (6.41 ng/mL), meaning that it has better luteal activity and can maintain pregnancy progesterone levels. In conclusion, Afzal Hussain and Hameed et al. (63) proved that MOLP supplementation significantly increased circulating progesterone levels during early gestation, which is an indicator of enhanced corpus luteum activity and luteal steroidogenesis. Over 8 days (breeding day 0) there was an augmentation of progesterone by approximately 20% (0.78 vs. 0.65 ng/mL), and by day 45 of the gestation period, levels had risen by approximately 94% in the high-MOLP group (6.41 vs. approximately 3.31 ng/mL; p ≤ 0.05).

Farrag et al. (59) evaluated the reproductive outcomes of Baladi goats supplemented with *Moringa oleifera* leaves from one month before mating through weaning and reported marked improvements in key reproductive indices. Farrag et al. reported that long-term supplementation with Moringa leaves increased kidding rate from 73.33 to 100% (+36%), litter size at birth by ~25% (1.47 vs. 1.18 kids/doe), and litter size at weaning by ~33% (1.33 vs. 1.00 kids/doe) (*p* < 0.01). Kid survival also improved, with mortality reduced by ~41% (9.09% vs. 15.38%), and weaning rate increased to 90.91%. Although hormones were not directly measured, these improvements strongly imply enhanced ovarian activity, ovulation rate, and embryonic survival linked to improved maternal nutritional and endocrine status.

Liang et al. (60) investigated the reproductive and endocrine responses of mature male cashmere goats to dietary supplementation with *M. oleifera* leaf powder (MOLP) and *M. oleifera* leaf ethanolic extract (MOLE), with particular emphasis on semen quality and reproductive hormone regulation. The circulating levels of gonadotropin-releasing hormone (GnRH) and testosterone were much better increased in the group of MOLE supplementation than in the control group (*p* < 0.05), which is evidence of a stimulatory action on the hypothalamic–pituitary-gonadal axis. These endocrine modifications were coupled with significant gains in the important reproductive performance parameters, such as the increase in sperm concentration, sperm motility, and sperm viability (all p < 0.05), which showed a direct functional expression of the hormonal modulation into better male fertility potential.

The study by Husien et al. (58) examined the impacts of *M. oleifera* leaf dietary inclusion (0, 20, 40, and 60%) on the reproductive performance, fertility characteristics, and gestation development in Nubian goats. Where moderate inclusion (20–40%) optimized fertility and fetal development, while excessive inclusion (60%) attenuated some responses. Litter size increased by up to ~15% (1.50 vs. 1.30; *p* < 0.001), gestation length extended by ~3 days, and fetal biometric indices were significantly enhanced (e.g., femur length ↑ ~ 31%, biparietal diameter ↑ ~ 8%). Birth weight responses were particularly notable: single male kids showed increases of ~16% (3.50 vs. 3.02 kg), and twin birth weights rose by ~53% relative to controls (p < 0.001).

**Figure 5:**
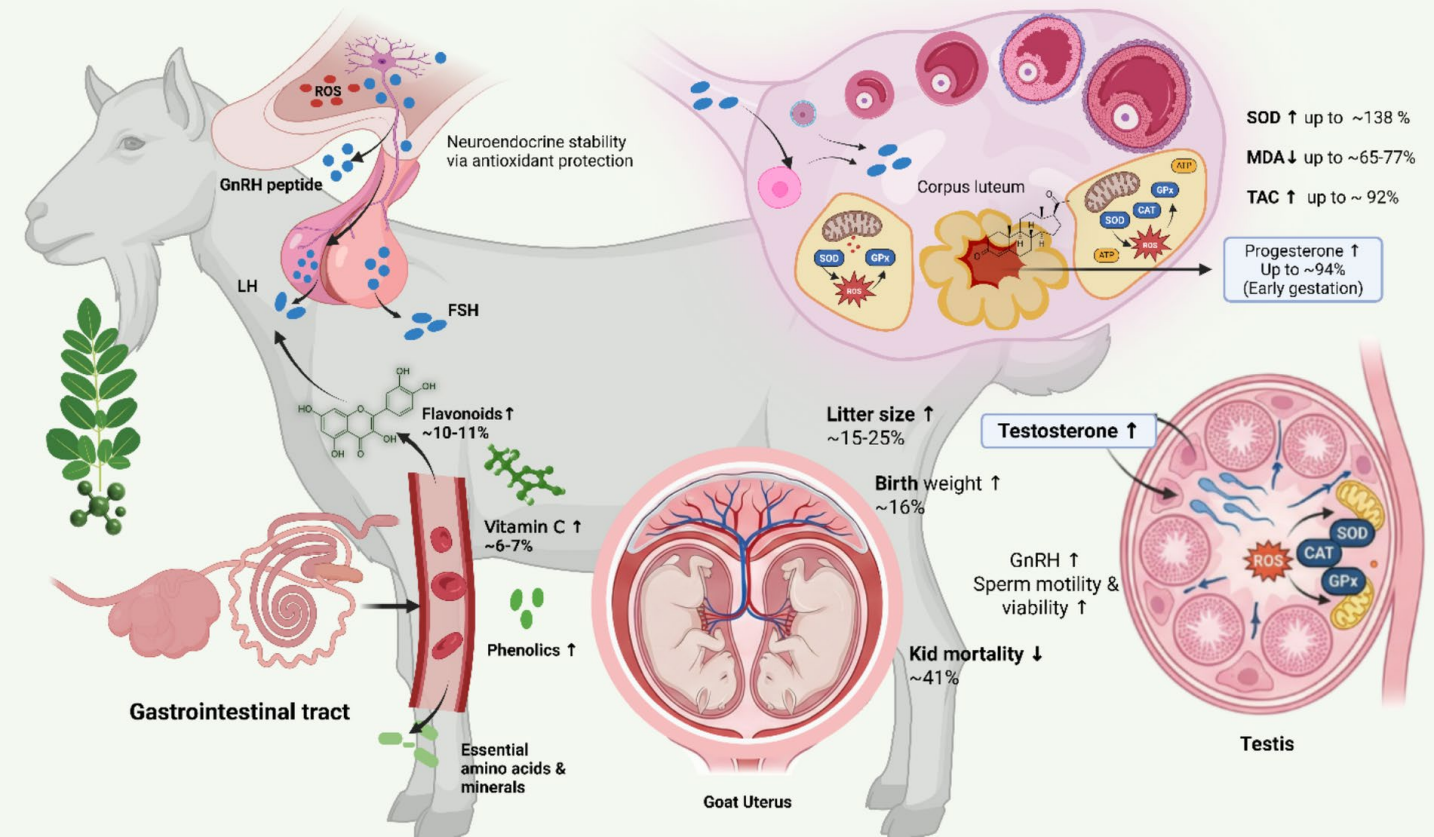
*Moringa oleifera* supplementation enhances reproductive and hormonal performance in goats (*Capra hircus*) through integrated cellular, endocrine, and antioxidant mechanisms. Dietary bioactives (flavonoids, phenolics, vitamin C, amino acids, minerals) elevate systemic antioxidant capacity, stabilizing hypothalamic–pituitary signaling (↑ GnRH, LH, FSH) and supporting gonadal function. In females, improved redox balance (↑ SOD, CAT, GPx; ↓ MDA; ↑ TAC) preserves mitochondrial integrity and promotes luteal steroidogenesis, increasing progesterone (up to ~94%), litter size (~15–25%), birth weight (~16%), and reducing kid mortality (~41%). In males, antioxidant protection of Leydig and germ cells enhances testosterone synthesis and spermatogenesis, collectively improving reproductive efficiency (BioRender).

On the mechanistic level, three processes that are closely connected (Figure 5) are involved in improving the reproductive and hormonal performance of the *M. oleifera* supplementation. To start with, there is stimulation of reproductive endocrine activity, manifested by significant increases in the levels of circulating reproductive hormones, such as progesterone levels (up to about 90% during early gestation) and increased GnRH and testosterone levels in males. It has been reported that there is improved testosterone production in relation to the nutrient contents of Moringa (such as Zn) in cattle and buffalo, which contribute to increased steroidogenic activity in the context of reproduction (104). The diets with *M. oleifera* leaf powder had effects on reproductive hormones in rabbits, raising progesterone in does with increasing doses and raising FSH/LH in bucks, but not testosterone, were inconsistent (105). The evidence from the review shows that *M. oleifera* leaf extracts have the potential to raise testosterone, FSH, and LH and improve sperm parameters in male rodent models by regulating reproductive endocrine and antioxidant pathways (106). *Moringa oleifera* is best described as a modulator of FSH-related reproductive function rather than a proven direct FSH stimulant. Its leaves and seeds contain polyphenols, flavonoids, vitamins, and other bioactive compounds with antioxidant activity, and these compounds can reduce oxidative stress while supporting reproductive endocrine function (107). In practical terms, this may help FSH regulation in three linked ways. First, by lowering reactive oxygen species, *Moringa* can protect hypothalamic–pituitary signaling, which is important because phytogenic antioxidants are recognized as a mechanism for improving reproductive efficiency in ruminants (108). Second, in heat-stressed female rabbits, *Moringa oleifera* supplementation was associated with higher serum FSH, together with LH, estradiol, progesterone, and prolactin, showing that it can improve gonadotropin profiles under stress conditions (109). Third, in Beetal goats, *Moringa* supplementation improved antioxidant defense and progesterone profile by suppressing reactive oxygen species, which suggests an indirect endocrine benefit that may help normalize reproductive hormone signaling (57). These hormonal changes represent an increase in corpus luteum activity, better steroidogenesis, and better provision of gametogenesis, which result in a more positive endocrine response to conception, embryo survival, and spermatogenic efficiency (103). Second, it leads to optimal maternal and fetal nutrition in the form of increased litter size of approximately 15–25% and increased birth weight by up to 16% in single offspring with moderate supplementation levels. Several of them have shown that *Moringa oleifera* leaf supplementation enhanced conception rate significantly and supported high multiple birth rates in heat-stressed Avishaan ewes, indicative of improved nutrient and reproductive status (105). Dietary supplement of *M. oleifera* leaf in mice enhanced litter size and litter birth weight in multiple gestations as compared to control, which was due to better nutrient status and antioxidant effects (110). Equally, in other species, such as New Zealand White rabbits, which were subjected to heat stress, co-treatment with *M. oleifera* seed extract alleviated stress-induced litter performance, and litter size and weight underwent a dose-dependent increase due to stress (109). *M. oleifera* supplementation improved colostrum protein status, antioxidant status, and the number of live-born piglets and enhanced biochemical indices in sows, which is a sign of improved nutrient status in progeny (90). Dietary addition of Moringa in early lactation Sahiwal cows has been shown to improve nutrient utilization, accelerate uterine involution, increase ovarian follicle numbers, and diminish reproductive disorders, which suggests improved metabolic and reproductive health, capable of supporting nutrient delivery to the growing fetus (111). In a study in the mouse Leydig TM3 cell model *in vitro*, mouse Leydig cells exposed to aqueous Leydig cell Leaf extract of *M. oleifera in-vitro*, glutathione (GSH) levels were elevated, and lipid peroxidation was inhibited, in addition to increased antioxidant response, which is direct cellular antioxidant activity associated with reproductive steroidogenic cells (112). The serum superoxide dismutase and total antioxidant capacity were significantly elevated in transition Holstein cows fed *M. oleifera* leaf meal (MOLM) than in the controls, indicating better oxidative stress protection during physiologically challenging times (113). Nonetheless, at inclusion levels that are too high, there is a diminution of some of the reproductive benefits, which suggests that physiological optimization, but not maximal dosing, is important. Taken together, these results show that *M. oleifera* is a reproductive nutraceutical useful in goats that incorporates endocrine regulation, enhanced nutrient metabolism, and provides defense against oxidative stress to boost fertility, prolificacy, and offspring performance (Table 5).

**Table 5:** Effect of *M. oleifera* on reproductive and hormonal performance.

Study	Species / breed	Supplementation strategy	Key reproductive outcomes (statistical values)	Hormonal outcomes (Statistical values)	Integrated interpretation	Ref
(Afzal Hussain and Hameed 2021)	Beetal goats (does)	MOLP at 1.6 and 3.2% of diet (60 days of gestation)	Conception maintained; reproductive efficiency supported indirectly via metabolic enhancement	Progesterone (ng/mL): Day 45 – Control = 5.45 ± 0.04, 1.6% = 5.72 ± 0.07, 3.2% = 6.41 ± 0.13 (*p* < 0.05)	Progesterone ↑↑ (dose-dependent luteal support enhancing pregnancy maintenance)	(57)
Farrag, 2024	Baladi goats	MOL leaves 50 g/doe/day (1 month pre-mating to weaning)	Kidding rate: 100 vs. 73.33%; Litter size at birth: 146.67 vs. 118.18; Weaning rate: 90.91 vs. 84.60% (*p* < 0.01)	Hormonal regulation inferred from improved fertility indices (no direct assay)	Reproductive efficiency ↑↑; kid mortality ↓↓; hormonal milieu ↑ (functional inference)	(59)
Liang et al., 2023	Goats (does)	*Moringa* supplementation during breeding–gestation	Estrus expression ↑; conception rate ↑; litter size ↑ (*p* < 0.05)	Progesterone and estradiol concentrations ↑ during luteal phase (*p* < 0.05)	Estrus response ↑; endocrine balance ↑ (improved ovarian function)	(60)
Husien et al., 2025	Nubian goats	0, 20, 40, 60% *M. oleifera* in roughage	Estrus response: 100% (20–40% vs. 80% control); Litter size: 1.50 (40%) vs. 1.30 (control); Birth weight: 2.75 kg vs. 1.95 kg (*p* < 0.05)	Gestation length ↑ (155.33 vs. 151.20 days, *p* < 0.05); fetal growth indices ↑	Fertility ↑↑ at moderate inclusion; fetal development ↑; excessive inclusion ↔/↓	(58)

MOLP: *M. oleifera* leaf powder; ↑: statistically significant increase (p ≤ 0.05); ↑↑: pronounced or dose-dependent increase; ↓: statistically significant decrease; ↓↓: marked or biologically favorable decrease; ↔: no statistically significant change; p: probability value; SEM: standard error of the mean.

### 4.4 Milk yield and milk quality attributes

The milk output and its compositional quality are the main economic variables of dairy goat businesses that directly influence the revenue from fluid milk and value-added products of cheese and yogurt. The composition of goat milk differs in terms of fat, proteins, and solids, which have a great impact on technological characteristics such as the yield of cheese and product (114, 115). These roles in market value are also fostered by a comprehensive profiling of milk composition (116). In new caprine systems, the most important quality determinants that set premium prices are milk output, fat and protein percentages, fatty-acid composition, and oxidative stability (117). Conventional rations tend not to maximize these parameters all at once; the high-concentrate diets are higher in yield at the expense of milk fat and more saturated fatty acids, and the forage-based systems are lower in yield at the expense of fat (118, 119). The present review synthesizes caprine-specific evidence across five lactation trials.

In lactating Anglo-Nubian goats, Kholif et al. (69) demonstrated that replacing sesame meal with Moringa leaf meal (MLM) significantly increased milk yield in a dose-dependent manner. Goats fed 15% MLM produced 943 g/day vs. 819 g/day in controls (~ + 15%; linear *p* = 0.0001). Energy-corrected milk increased to 917 g/day, and milk energy output reached 2.93 MJ/day (*p* < 0.001). Milk component yields were markedly enhanced: protein output increased by ~17% (36.0 vs. 30.8 g/day), and lactose output by ~26% (*p* < 0.0001). Milk lipid quality also improved, with CLA rising from 0.68 to 1.07% (~ + 57%; *p* = 0.0004) and the UFA/SFA ratio increasing from 0.392 to 0.572 (*p* = 0.0014), indicating improved functional milk fat quality.

Similar production responses were reported by Kholif et al. (62) when berseem clover was replaced with *Moringa* leaves in Nubian goats. Milk yield increased from 809 to 900 g/day (~ + 11%, linear *p* < 0.01), while ECM rose from 705 to 836 g/day (~ + 19%). Total solids yield increased by ~17%, fat yield by ~25%, protein by ~14%, and lactose by ~13%. Milk efficiency (ECM/DMI) improved from 0.89 to 0.98 (*p* = 0.02), confirming better conversion of feed nutrients into milk. Fatty acid profiles shifted toward higher MUFA, PUFA, and CLA with reduced SFA and lower atherogenicity index (2.32 → 1.91), indicating healthier milk lipid composition. During the transition to lactation, Afzal et al. (63) showed that MOLP supplementation significantly elevated early-lactation milk yield in Beetal goats. By day 28, milk production increased from 1.87 L (control) to 2.72 L in the 3.5% MOLP group (~ + 45%, *p* = 0.04), demonstrating strong responsiveness during periods of high metabolic demand. These improvements likely reflect enhanced energy balance, antioxidant support, and nutrient partitioning toward lactation.

Under semi-intensive conditions, Farrag et al. (59) found that supplementation with 50 g *Moringa* leaves/day increased milk yield from 802 to 961 g/day (~ + 20%, *p* = 0.003) and ECM from 465 to 671 g/day (*p* < 0.001). Fat yield increased by ~43% and protein yield by ~47%, while total solids rose significantly. These responses indicate enhanced mammary synthesis supported by improved rumen fermentation and metabolizable energy supply. While most studies show yield increases, Cohen-Zinder et al. (67) demonstrated that *Moringa* silage primarily enhanced milk quality rather than volume (milk yield *p* > 0.05). Milk protein, lactose, and fat contents increased (*p* < 0.05), somatic cell count declined, antioxidant capacity increased, and MDA concentrations fell to 20.8–22.4 nmol/mL (*p* < 0.05), indicating improved oxidative stability. Milk phospholipids and n-3 fatty acids increased, and the n-6/n-3 ratio improved (7.2 → 5.2; *p* < 0.01), demonstrating enhanced nutritional and technological milk quality.

At a mechanistic level, the improvements in milk yield and milk quality associated with *M. oleifera* supplementation arise from three tightly interconnected physiological processes involving rumen metabolism, mammary nutrient supply, and antioxidant-mediated milk stabilization. First, enhanced nutrient availability and mammary substrate supply form the primary production-driving mechanism. Across studies, dry matter intake and digestible nutrient supply increased by approximately 8–12%, while energy utilization efficiency improved by about 6%, resulting in greater delivery of metabolizable protein, acetate, and glucogenic precursors to the mammary gland. An *in vitro* batch culture study reported that inclusion of *M. oleifera* leaves up to 10–20% in goat rumen fluid increased true dry matter and organic matter digestibility, microbial biomass, and concentrations of acetate and propionate (key volatile fatty acids) compared to control fermentation conditions. This indicates enhanced nutrient availability and microbial activity (55). Similarly, when *M. oleifera* leaf silage replaced soybean meal in an *in vitro* fermentation system, total gas production kinetics, acetate, and propionate concentrations increased, pointing to greater fermentative breakdown of substrates and enhanced availability of glucogenic and lipogenic precursors (114). *In Vivo*, findings also gave similar outcomes. For example, *M. oleifera* inclusion in diets improved dry matter intake and digestibility in dairy cattle fed basal forage diets (115). These shifts translated into milk yield increases of ~11–45% depending on physiological stage, with energy-corrected milk improving by ~15–20%. This increase is also evidenced by bovine studies. In lactating multiparous Holstein dairy cows, feeding a *M. oleifera* rachis and twig preparation at 6% of diet increased milk yield and milk fat, compared to control diets (120). Lactating Friesian cows supplemented with *M. oleifera* leaves showed significantly higher actual milk yield and 4% fat-corrected milk yield compared with unsupplemented controls (118).

Second, stimulation of mammary synthetic activity enhanced the secretion of major milk constituents. Protein yield increased by approximately 14–47%, fat yield by 25–43%, and lactose yield by 13–26%, contributing to total solids increases of ~15–17%. In lactating buffaloes, *M. oleifera* leaves powder supplementation significantly increased milk protein, fat %age, and total solids yield (g/day) compared with controls, evidence of enhanced mammary synthetic activity (93). Review literature notes that *M. oleifera* supplementation in dairy ruminants (cows, goats) often leads to increases in milk constituents, including protein, fat, and total solids, linked to improved nutrient supply and rumen fermentation metabolism relevant to mammary synthesis (33). Third, phytochemical transfer and antioxidant-mediated stabilization of milk lipids improved the functional quality of milk. Supplementation increased milk unsaturated fatty acids and CLA (up to ~57% higher), elevated n-3 fatty acids, and reduced saturated fatty acids and atherogenicity index (↓ ~ 18%). Feeding *M. oleifera* rachis and twig preparation to lactating dairy cows increased total unsaturated fatty acids (UFAs), mono- and polyunsaturated fats, including n-3 fatty acids in milk compared to controls, indicating improved milk lipid quality (120). Supplementation of *Moringa* essential oils in lactating sheep increased proportions of UFAs, MUFAs, and total CLA, and improved UFA: SFA ratios, consistent with antioxidant-related modulation of ruminal biohydrogenation (119). Concurrently, oxidative stability improved, with milk malondialdehyde reduced and antioxidant capacity elevated, indicating protection of milk lipids from peroxidation. Somatic cells count also declined, reflecting improved mammary health and immune stability. The integrated effects of *M. oleifera* on Milk Yield and Milk Quality Attributes are shown in (Table 6).

**Table 6:** Effect of *M. oleifera* on milk yield and milk quality attributes.

Study	Moringa form and level	Milk yield response	Milk component yields (g/day)	Milk composition (g/kg unless stated)	Fatty acid/lipid quality changes	Other milk quality indicators	Ref
Kholif et al., 2015 (Anglo-Nubian)	Leaf meal replacing sesame meal: 0, 10, 15, 20% of diet DM	↑ Highest at M15: 943 g/d vs. 818.6 g/d control (*p* < 0.0001; linear *p* = 0.0001)	Fat: 33.36 vs. 29.05 (*p* = 0.0002) Protein: 36.00 vs. 30.84 (*p* < 0.0001) Lactose: 43.09 vs. 34.19 (*p* < 0.0001)	Total solids ↑ 126.3 vs. 122.0 (*p* = 0.0402) Lactose ↑ 45.7 vs. 41.6 (*p* = 0.001)	TSFA ↓ 71.89 → 63.65% (*p* = 0.001) MUFA ↑ 26.98 → 34.76% (*p* = 0.0014) PUFA ↑ (*p* = 0.0051) CLA ↑ 0.68 → 1.07% (*p* = 0.0004) TUFA/TSFA ↑ (*p* = 0.0014)	Improved milk energy output (2.47 → 2.93 MJ/d; *p* < 0.0001)	(69)
Kholif et al., 2017 (Nubian)	Fresh MO replacing berseem clover: 0, 12.5, 25, 37.5%	↑ 809 → 900 g/d (*p* = 0.01; linear *p* < 0.01)	Fat: 24.9 → 31.1 (*p* < 0.01) Protein: 28.7 → 32.8 (p = 0.02) Lactose: 33.1 → 37.4 (*p* = 0.04)	TS ↑ 114.1 → 119.3 (*p* = 0.03) Fat ↑ 30.9 → 34.6 (*p* < 0.01)	SFA ↓ 70.4 → 66.7% (*p* < 0.01) UFA ↑ 29.6 → 33.3% (*p* < 0.01) MUFA ↑ (*p* < 0.01) PUFA ↑ (*p* = 0.01) CLA ↑ 0.69 → 0.88% (*p* = 0.02) AI ↓ 2.32 → 1.91 (*p* < 0.01)	ECM efficiency ↑ (0.89 → 0.98; *p* = 0.02)	(62)
Afzal et al., 2022 (Beetal)	MOLP 0, 2, 3.5% (5–8.75 g/d)	↑ Dose-dependent: Day 28: 1.87 → 2.72 L/d (*p* = 0.04)	Not reported as g/d, but overall production ↑	Composition not detailed	Not reported	Early lactation performance improved	(63)
Farrag, 2024 (Baladi)	MOL leaves 50 g/doe/day	↑ 802 → 961 g/d (*p* = 0.003) ECM ↑ 465 → 671 g/d (*p* < 0.001)	Fat: 23.5 → 33.5 (*p* = 0.010) Protein: 23.2 → 34.2 (*p* = 0.001)	Fat ↑ 29.3 → 34.9 (*p* = 0.001) Protein ↑ 28.9 → 35.4 (*p* = 0.001) Lactose ↑ (*p* = 0.027) TS ↑ 103.5 → 120.2 (*p* < 0.001)	FA profile not given	MEC numerically ↑ (*p* = 0.057)	(59)
Cohen-Zinder et al., 2025 (Dairy goats)	Moringa silage replacing forage	Milk yield maintained (*P* > 0.05)	Not primary outcome	Fat ↑ (wk 8; *P* < 0.05) Protein ↑ (wk 4; *p* < 0.05) Lactose ↑ 4.58% (*p* < 0.05)	n-3 FA ↑ (*p* < 0.01) n-6/n-3 ↓ 7.2 → 5.2 (*p* < 0.01) Phospholipids ↑ (*p* < 0.05–0.001)	SCC ↓ (*p* < 0.05) MDA ↓ 20.8–22.4 nmol/mL (*p* < 0.05) *α*-tocopherol ↑	(67)

## 5 Conclusion and future perspectives

All the facts prove that *M. oleifera* is a versatile phytogenic feed additive, increasing the efficiency of metabolism, physiological stability, and productive functioning of goats. The moderate nutritional inclusion of the diet led to a consistent increase in nutrient utilization, as denoted in the nitrogen balance improvement of about 25% and the increase in fibre digestibility of up to around 8%, indicating improved efficacy of rumen fermentation and nutrient capture. These metabolic advantages were coupled with significant improvement of systemic antioxidant defenses with enzyme activities like the superoxide dismutase rising by over 100% in certain physiological phases, and the lipid peroxidation decreasing by up to approximately 70%, showing a significant switching to oxidative activity and balances in the instances of high metabolic rates. The embryo was endocrinologically and developmentally stimulated to alter the reproductive performance in a positive manner. Optimal supplementation of progesterone levels during early gestation were associated with almost 90% increases in litter size (≈15–25%), and higher birth weights in singles (to almost 16%), indicating improved luteal functioning, placental performance, and nutrient displacement of the mother. Lactation reactors were higher milk production and production of milk components and better milk fatty acid composition, in terms of increased unsaturated fatty acids and an increased oxidative stability indicating that Moringa also augments the milk nutritional and functional properties. Mechanically, they are the result of the coordination of three fundamental processes, which are enhanced nutrient provision and rumen efficiency, endocrine-assisted reproduction, and strong antioxidant-mediated cell protection. Notably, the most stable benefits were observed in moderate inclusion level, but at excessive supplementation, some responses were suppressed, instead of maximizing dosing but physiological optimization. Future studies are aimed at identifying the specific dose–response relationships between the breeds and production systems, defining the rumen microbiome responses through the use of omics methods, and the eventual clarification of the molecular pathways and Moringa phytochemicals to endocrine and redox regulation. Translating *M. oleifera* as a nutraceutical into a standardized part of climate-resilient small ruminant production systems will rely heavily on long-term research on the referenced carryover effects on offspring performance, milk processing properties and economic sustainability.

## Data Availability

The original contributions presented in the study are included in the article/supplementary material, further inquiries can be directed to the corresponding authors.
